# Basal Cell Carcinoma of the Right Lower Eyelid Treated With Tarsoconjunctival and Mustardé Flaps

**DOI:** 10.7759/cureus.79129

**Published:** 2025-02-16

**Authors:** Ilias Anastasopoulos, Aristeidis Ntamagkas, Dimosthenis Chrysikos, Andreas Patelis, Theodore Troupis

**Affiliations:** 1 Department of Anatomy, Medical School, National and Kapodistrian University of Athens, Athens, GRC; 2 Plastic Surgery Clinic, 251 Air Force General Hospital, Athens, GRC; 3 Oculoplastic Clinic, 251 Air Force General Hospital, Athens, GRC

**Keywords:** basal cell carcinoma, hughes flap, lower eyelid defect, mustardé flap, tarsoconjunctival flap

## Abstract

An 82-year-old female patient presented with noduloulcerative basal cell carcinoma (BCC) on the right lower eyelid, extending laterally. Following full-thickness excision of the carcinoma with clear margins, a tarsoconjunctival flap was placed over the posterior lamella to create support and lining. A Mustardé flap was used for anterior lamella repair, and a rotating cheek flap was used to recreate the natural shape and function of the eyelid. Postoperative recovery was uneventful, with excellent flap viability and minimal complications. At follow-up, the patient demonstrated well-aligned eyelid function, good aesthetic outcomes, and histopathological confirmation of complete tumor excision. Hughes and Mustardé flaps combined in a single-step procedure offer a reliable and effective solution for managing extensive full-thickness eyelid defects post-BCC excision, as it maintains eyelid integrity, minimizes morbidity, and achieves favorable cosmetic and functional outcomes, serving as an invaluable approach in complex periocular reconstructions.

## Introduction

Basal cell carcinoma (BCC) is the most common malignant tumor, affecting the periocular region and accounting for approximately 90% of all eyelid cancers. It typically occurs in older patients exposed to ultraviolet radiation, often localized on the lower eyelid due to its direct exposure to sunlight [1-4]. Despite being locally invasive, BCC rarely metastasizes but can cause significant destruction of surrounding tissues if not treated promptly [4-7]. Reconstructive surgery following tumor excision presents a unique challenge, especially for full-thickness defects that require restoration of both functional and aesthetic properties of the eyelid [5,6,8].

The Hughes tarsoconjunctival flap is a widely accepted technique for reconstructing posterior lamella defects, providing critical tarsal support and conjunctival lining to restore the stability of the lower eyelid [5,9-11]. This treatment, often used in cases with significant tissue loss, is highly effective for lower eyelid reconstruction due to its robust vascularization. It minimizes the risk of flap dehiscence and complications [8]. The Mustardé flap provides excellent skin coverage and structural support using a rotational cheek flap that blends well with adjacent tissue, achieving functional and aesthetic continuity [1-3,11].

Combining the Hughes flap with the Mustardé cheek rotation flap in significant, full-thickness defects can offer enhanced outcomes [12]. Combining these two flaps addresses the multi-layered complexity of full-thickness eyelid defects. The Hughes flap’s solid tarsoconjunctival reconstruction secures the posterior lamella. In contrast, the Mustardé flap’s anterior coverage optimally recreates the skin and muscle layers needed for a natural appearance and eyelid mobility [4,7,9]. This dual approach leverages the rich vascular network of the facial tissues, supporting faster healing and reducing the risk of postoperative complications such as ectropion, lid retraction, or inadequate structural support, which are concerns with other reconstructive methods [5,10,11].

## Case presentation

**Figure 1:**
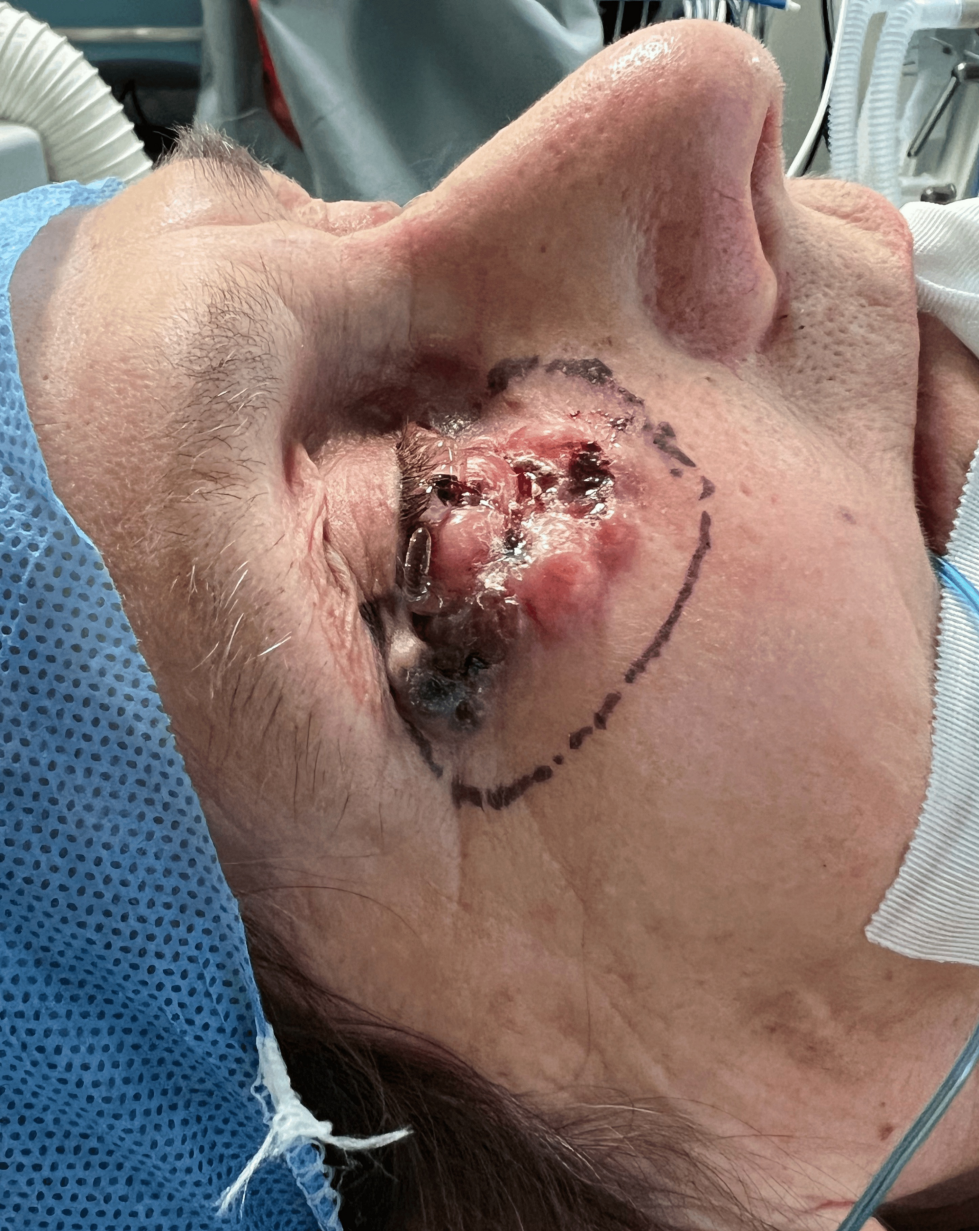
Full-thickness defect of the lower eyelid. The defect is demarcated with a surgical marking pen, and evidence of tissue damage and inflammation is visible.

An 82-year-old female patient presented with an ulcerative lesion located on the right lower eyelid, near the medial canthus of the eye, on the lower eyelid, extending to the lateral canthus and the adjacent cheek area (Figure 1).

The lesion was notably ulcerated, with an irregular surface. The edges of the ulcerated area appeared to be raised, creating a crater-like appearance, with the central ulcerated portion. The lesion showed areas of crusting, likely from dried exudate or blood, and some signs of minor bleeding. The margins were poorly defined but marked with preoperative surgical lines (in purple ink), indicating a plan for excision with clear margins.

Diagnostic evaluation

The lesion’s overall appearance suggested a noduloulcerative subtype of BCC, characterized by a combination of raised edges and central ulceration [13]. There was no involvement of the upper eyelid, and no regional lymphadenopathy was noted. Other considerations include squamous cell carcinoma (SCC), which can present similarly but typically has more of a scaly or keratotic appearance [14]. Before surgery, imaging assessments revealed no signs of orbital invasion, with no indication of deep structure involvement or distant metastases. A postoperation biopsy validated the nodular BCC diagnosis.

Surgical procedure

Due to the lesion's location and size, surgical removal was scheduled under general anesthesia, ensuring the best patient comfort and surgical precision. The margins were meticulously defined to ensure oncological safety. The goal was to excise the lesion with clear margins while maintaining the functional integrity of the eyelid and preparing for subsequent reconstruction. The surgical steps are explained below.

Excision of the Tumor

**Figure 2:**
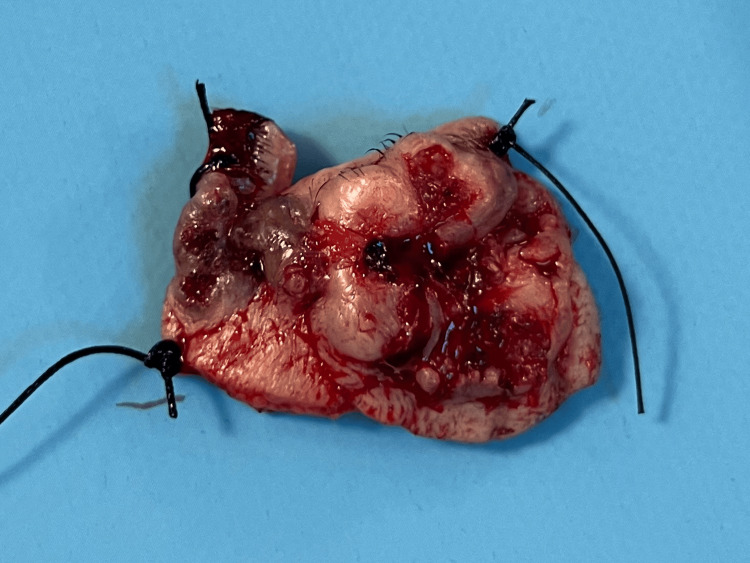
Excised specimen marked in order to undergo histopathological analysis to confirm the diagnosis.

The carcinoma was excised with a 5 mm margin to ensure precise resection, resulting in a full-thickness defect involving the entire height of the lower eyelid. The skin, subcutaneous tissue, and a portion of the orbicularis oculi muscle were removed. The defect extended to the lateral canthal area. The excised specimen was sent for frozen section analysis to confirm that clear surgical margins were achieved (Figure 2).

**Figure 3:**
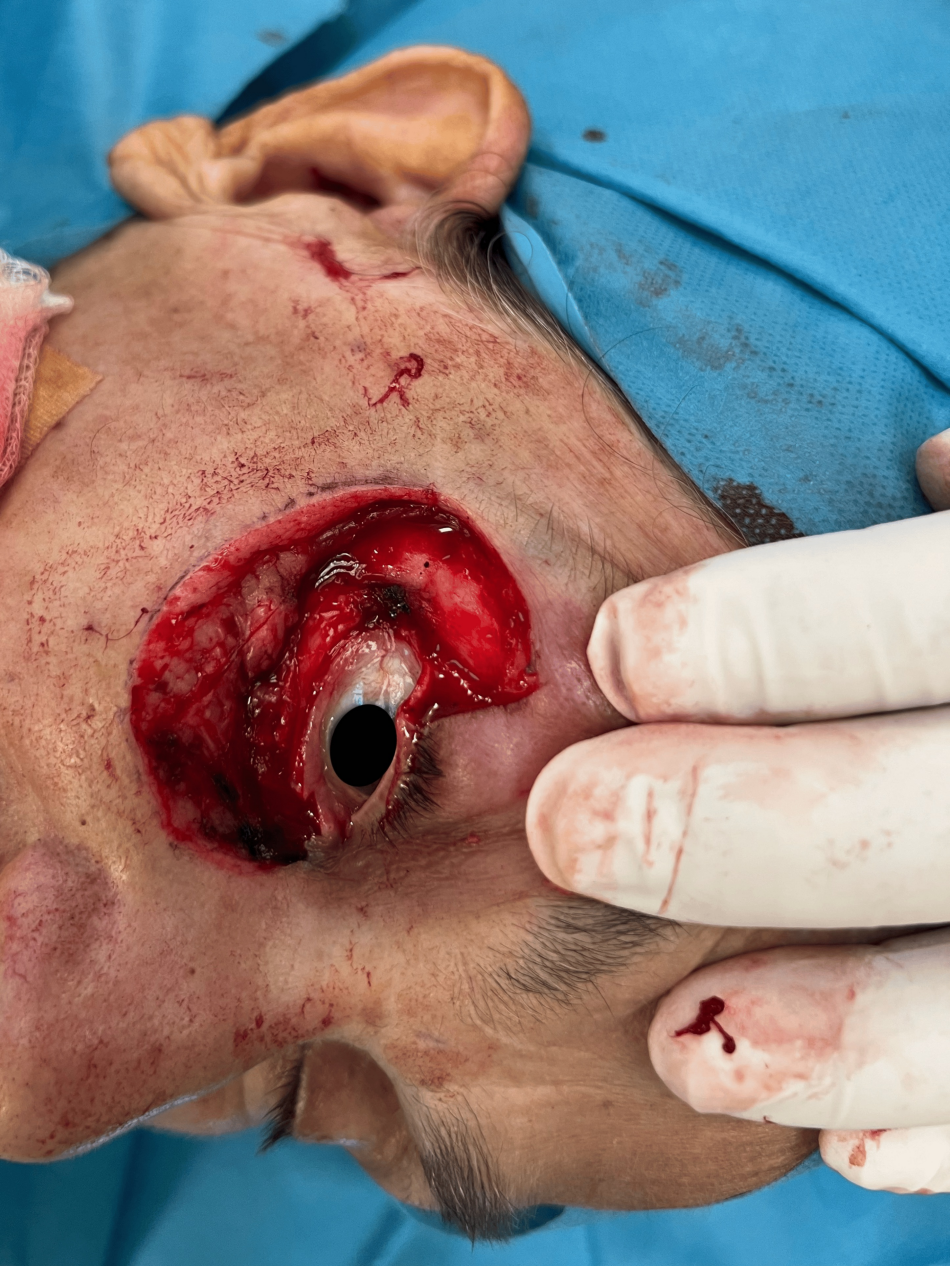
Intraoperative photograph demonstrating the surgical site following excision of a full-thickness lower eyelid defect.

The wound's edges were well-defined, and careful hemostasis was achieved to control bleeding from the excised muscle and soft tissues (Figure 3).

Reconstruction with Tarsoconjunctival Flap

**Figure 4:**
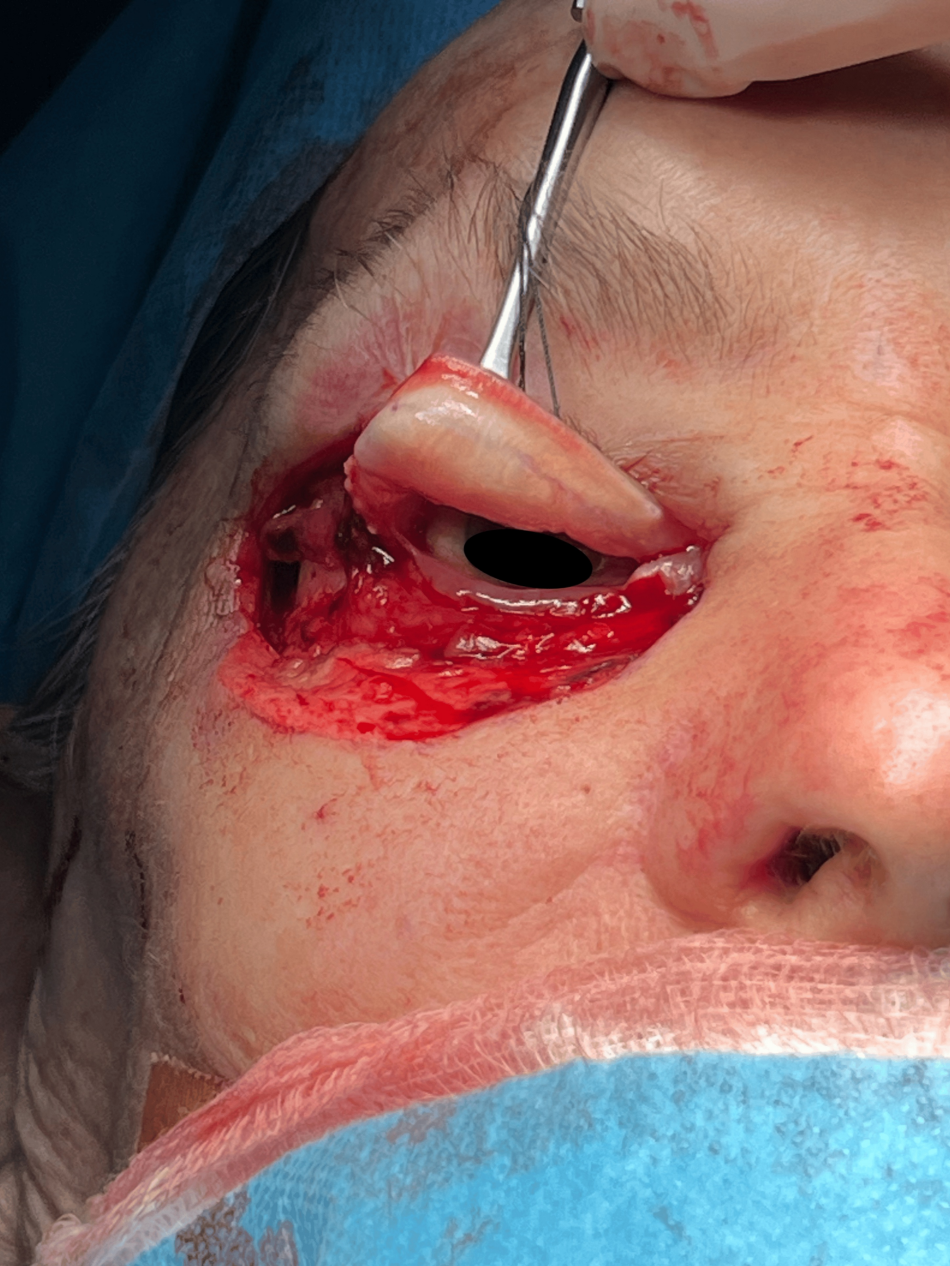
The tarsoconjunctival layer is being carefully retracted with surgical forceps in prder to reconstruct the posterior lamellar defect using a tarsoconjunctival flap harvested from the ipsilateral upper eyelid.

The posterior lamellar defect was reconstructed using a tarsoconjunctival flap harvested from the ipsilateral upper eyelid (Figure 4).

**Figure 5:**
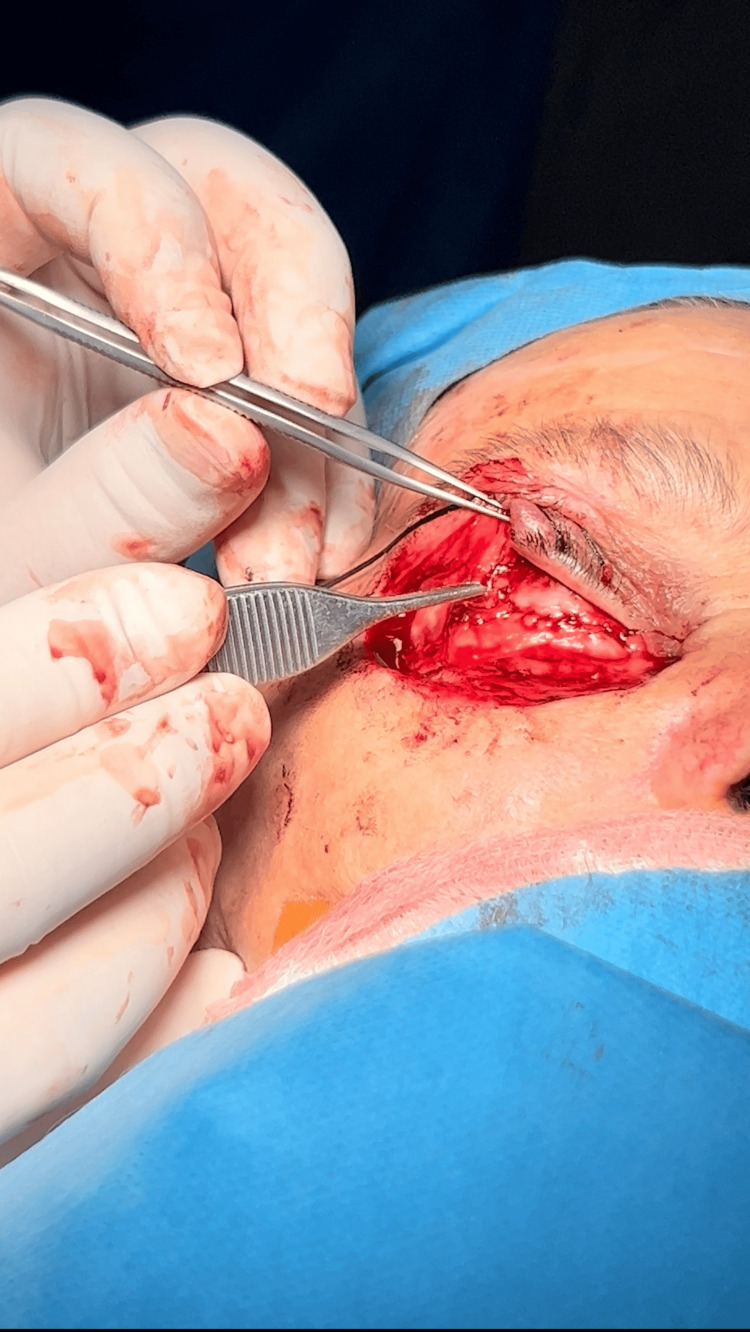
The conjunctival surface is being sutured to the residual conjunctiva of the lower eyelid.

An incision was made along the tarsal plate and conjunctiva of the top eyelid to form a flap. The flap was elevated, maintaining its vascular supply to guarantee its viability. The flap was precisely positioned to match the lower eyelid's structure and conceal the defect caused by the resection. The conjunctival surface was sutured to the residual conjunctiva of the lower eyelid to establish a continuous inner lining (Figure 5).

 The flap remained positioned for three weeks and then separated from the upper lid.

**Figure 6:**
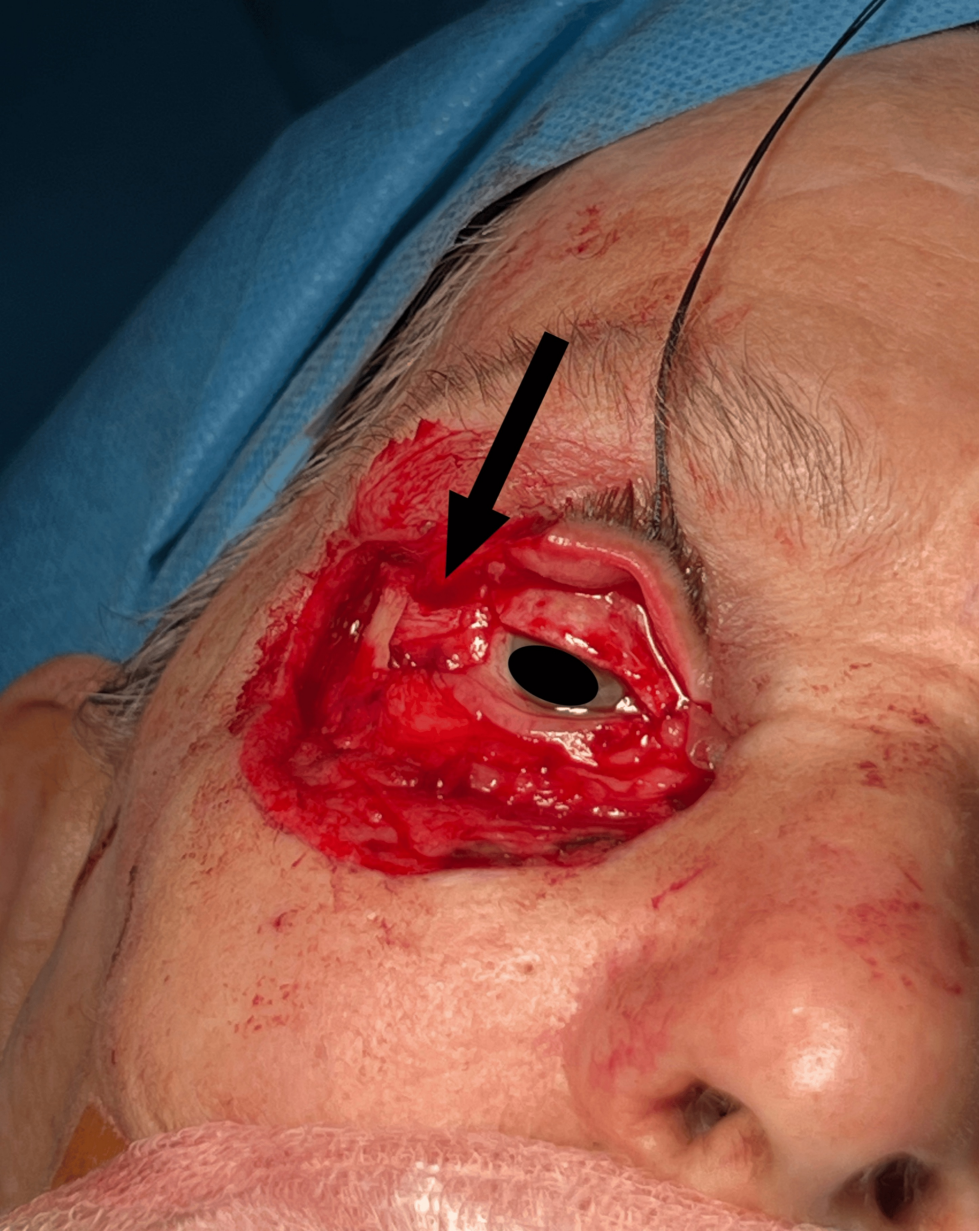
The black arrow indicates the shortened lateral canthal tendon which is repositioned to reinforce the lower eyelid.

Given the significant tissue loss and the need to maintain eyelid function and normal contour, a canthoplasty was performed. The lateral canthal tendon was shortened and repositioned to reinforce the lower eyelid, ensuring proper eyelid function and maintaining the integrity of the ocular surface. The tarsal plate was secured with the lateral canthal tendon to the periosteum of the lateral orbital rim, stabilizing the eyelid and preventing ectropion (outward turning of the eyelid) (Figure 6).

Reconstruction with Mustardé Flap

**Figure 7:**
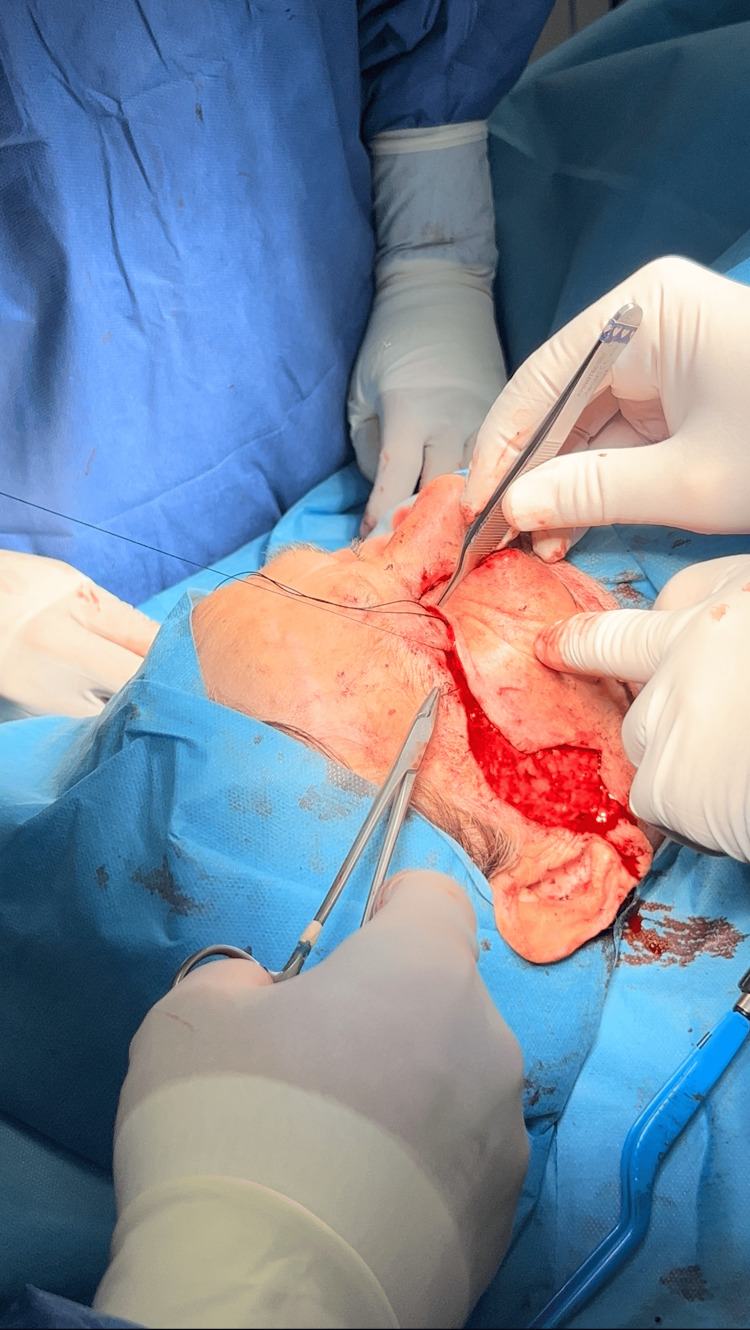
The rotation of the Mustardé flap in order to restore the deficit.

After the tarsoconjunctival flap reconstruction, a Mustardé flap was performed to complete the defect's external closure and restore the eyelid's entire structure. The Mustardé flap was meticulously dissected while maintaining its blood supply (subdermal vascular plexus), which is essential for its viability and recovery. The skin and underlying tissue were elevated from the donor site, preserving a pedicle to the adjacent skin to guarantee adequate blood supply to the flap. The Mustardé flap was then rotated into the lower eyelid defect, ensuring it perfectly matched the area's dimensions needing coverage (Figure 7).

**Figure 8:**
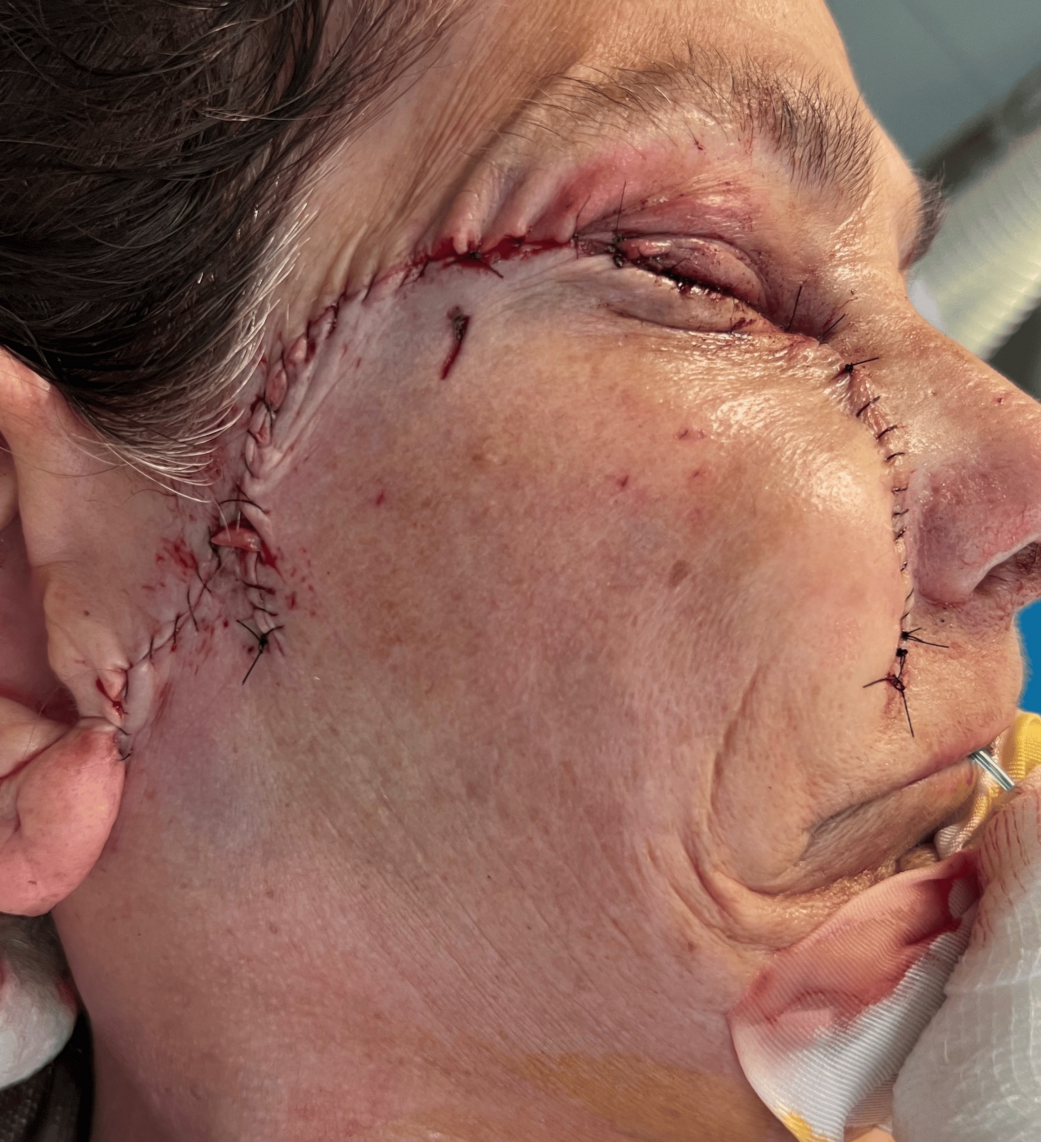
Immediate postoperative lateral view following reconstruction of a full-thickness lower eyelid defect. The image highlights precise surgical closure and alignment of the tissues to achieve optimal functional and cosmetic outcomes. Early postoperative edema and erythema are noted, consistent with the healing process.

The flap was placed over the tarsoconjunctival flap, supplying the external dermal layer and aiming for a natural appearance of the eyelid. The flap's borders were sutured to the adjacent skin and muscle layers of the lower eyelid without tension in order to preserve the natural curve of the eyelid and cheek. Upon securing the flap, the donor location on the face, from which the skin was excised, was also sutured. The cheek incision was conventionally sutured in layers to minimize strain and avert scarring or abnormalities in the region. Final modifications were made in order to maintain a natural eyelid contour and preserve eyelid function (Figure 8).

Postoperative Care and Outcome

Postoperatively, the patient was admitted for observation to monitor for flap viability, infection, and proper wound healing. The eye was covered with a light dressing, and the patient was prescribed systemic antibiotics and topical lubricants.

At the one-week follow-up, the flaps were viable with no signs of necrosis or infection. The patient had minimal swelling and bruising, which resolved over subsequent weeks. The reconstructed eyelid showed good alignment and function, with excellent cosmetic results. Histopathological analysis of the excised tissue confirmed complete excision of the carcinoma with clear margins.

**Figure 9:**
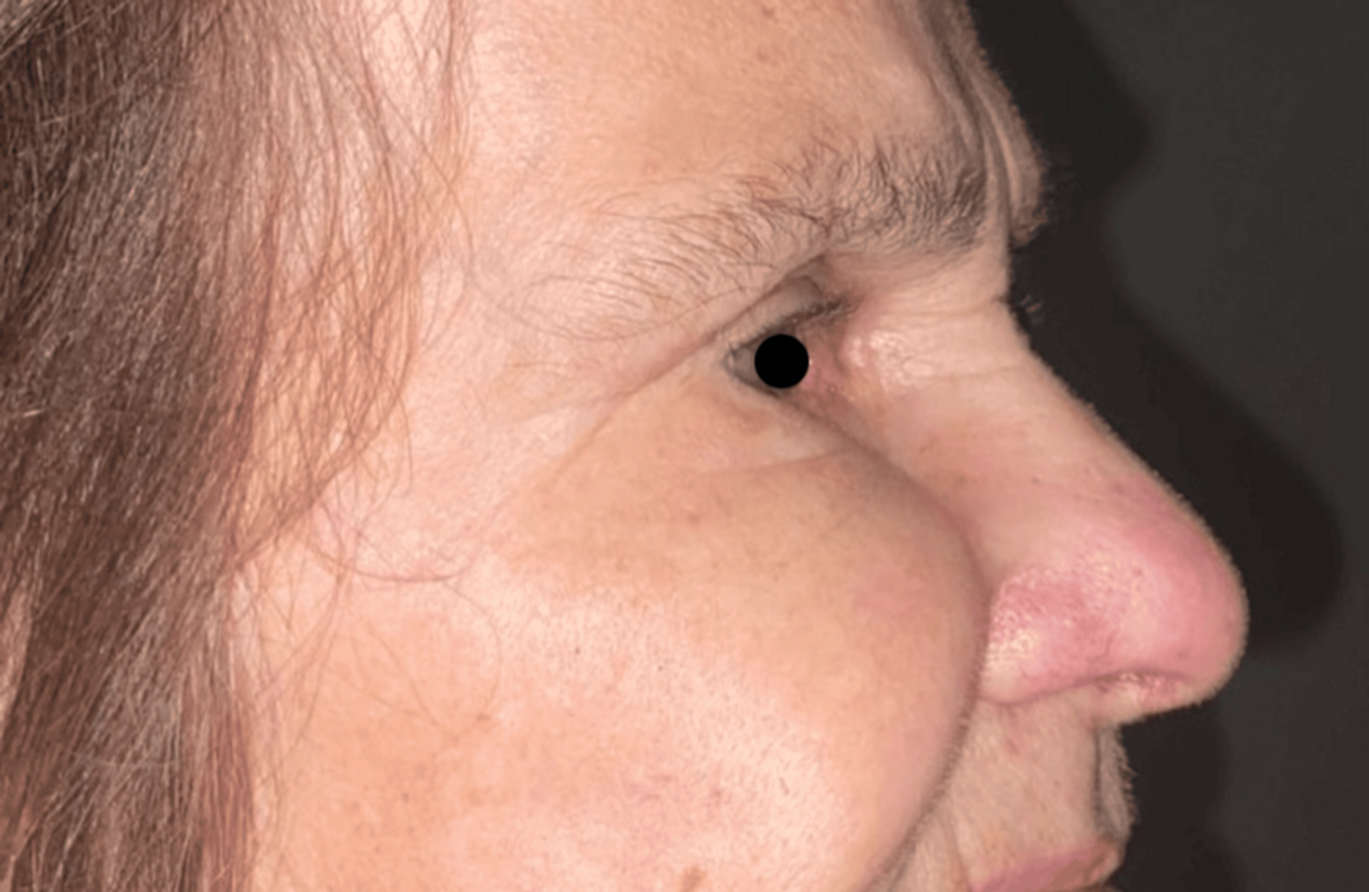
The absence of significant deformity or visible scarring further emphasizes the success of the surgical procedure in achieving both functional and aesthetic outcomes. The authors have written informed consent from the patient to publish images of the patient's face in this article in this open-access journal

**Figure 10:**
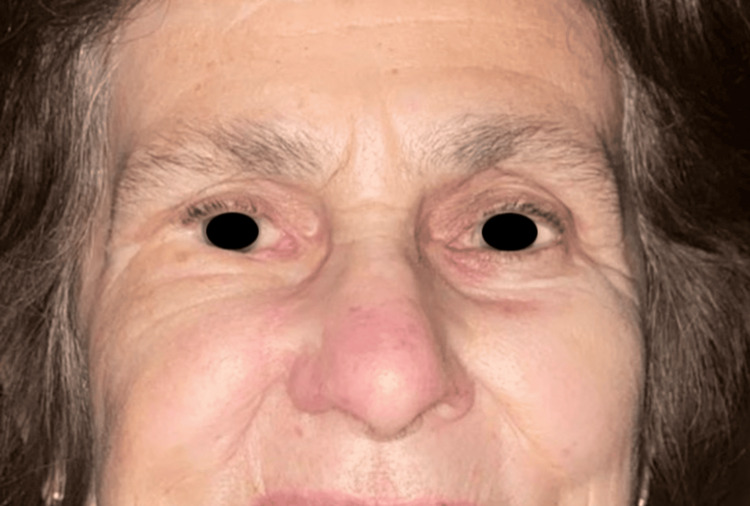
Postoperative photograph taken six months after surgical reconstruction of a full-thickness lower eyelid defect. The result demonstrates excellent restoration of eyelid contour, symmetry, and functionality. The authors have written informed consent from the patient to publish images of the patient's face in this article in this open-access journal

The postoperative outcome at six months demonstrated an impressive level of both functional and aesthetic success. (Figures 9, 10). The reconstructed lower eyelid exhibited remarkable symmetry, seamlessly integrating with the surrounding facial structures. The alignment of the eyelid margin was precise, ensuring optimal protection of the globe and maintaining the natural curvature and contour of the eyelid. Functionally, the eyelid performed its roles effectively, with no evidence of ectropion, lid retraction, or other complications that often arise in complex reconstructions. The restoration of the tear film distribution and ocular surface integrity confirms the functional viability of the reconstruction.

Aesthetically, the surgical site healed with minimal scarring, and the use of adjacent vascularized tissue has achieved a natural skin tone and texture that blended harmoniously with the adjacent areas. This outcome reflects meticulous surgical planning and execution, underscoring the efficacy of the combined Hughes and Mustardé flaps in addressing full-thickness lower eyelid defects.

## Discussion

Reconstructing full-thickness lower eyelid defects following BCC excision presents a distinct challenge due to the eyelid's complex functional and aesthetic roles. BCC, the most common malignant tumor in the periocular region, has a high incidence in the lower eyelid due to ultraviolet exposure, necessitating effective reconstructive strategies to preserve stability and appearance [2,11]. Although various methods exist, the combined use of Hughes and Mustardé flaps offers a comprehensive solution that is particularly valuable in large or complex defects where single-layer reconstruction would be insufficient [8,15].

The Hughes tarsoconjunctival flap reconstructs the posterior lamella with reliable support from the upper eyelid’s tarsal and conjunctival tissues, maintaining the internal integrity necessary for eyelid stability and ocular protection. However, as it solely addresses the internal structure, combining it with an anterior lamellar reconstruction is essential in cases requiring skin and orbicularis muscle restoration [9,16]. In contrast, the Mustardé cheek rotation flap provides robust vascularized coverage for the anterior lamella, seamlessly blending with the surrounding facial tissue to create a natural contour that complements the posterior reconstruction [11,12].

Using Hughes and Mustardé flaps in tandem facilitates a full-thickness approach that addresses both lamellae, leveraging the vascular supply from adjacent facial regions. This dual-layered reconstruction promotes faster healing, reduces flap-related complications such as ischemia, and decreases ectropion and lid retraction risks, which are common concerns in lower eyelid surgeries [1,5]. As outlined in Grabb and Smith’s Plastic Surgery, this method embodies the principle of “replacing like with like,” a key concept in achieving both form and function in complex reconstructions [16].

This case report's clinical relevance lies in demonstrating an underutilized but effective approach for extensive lower eyelid defects, especially as the simultaneous application of Hughes and Mustardé flaps remains underreported. By providing practical insights into the benefits of this combined technique, this report contributes to the body of knowledge in periocular reconstruction. It may inform future treatment strategies for similarly challenging cases [4,7].

## Conclusions

This case report demonstrates the effective use of a combined Hughes and Mustardé flap technique in reconstructing a significant, full-thickness defect of the lower eyelid following basal cell carcinoma excision. The Hughes flap provided critical support for the posterior lamella, while the Mustardé flap addressed the anterior lamella, achieving an optimal balance of structure, function, and aesthetics. The successful outcome of this approach, with minimal complications and excellent functional and cosmetic results, underscores the viability of using these flaps together in a single-stage procedure.

The combined Hughes and Mustardé technique presents a valuable alternative to traditional multi-stage reconstructions, offering reduced recovery times and fewer complications, particularly for large defects involving both eyelid lamellae. Given the frequent occurrence of BCC in the periocular region and the complex requirements of eyelid reconstruction, this report contributes to the growing body of knowledge on periocular reconstructive options and may guide future surgical practices in managing extensive eyelid defects. The publication of this case serves to inform and inspire clinicians on effective techniques for challenging reconstructions, ensuring that patients receive both functional integrity and aesthetic satisfaction in their treatment outcomes.
